# Supplementary material for: Degeneracy in the neurological model of auditory speech repetition
Source: Commun Biol. 2023 Nov 13;6:1161. doi: 10.1038/s42003-023-05515-5 (PMC10643365; doi:10.1038/s42003-023-05515-5)
Supplement: Supplementary file 2 — Supplementary Information [file 42003_2023_5515_MOESM2_ESM.pdf]

Supplementary Information

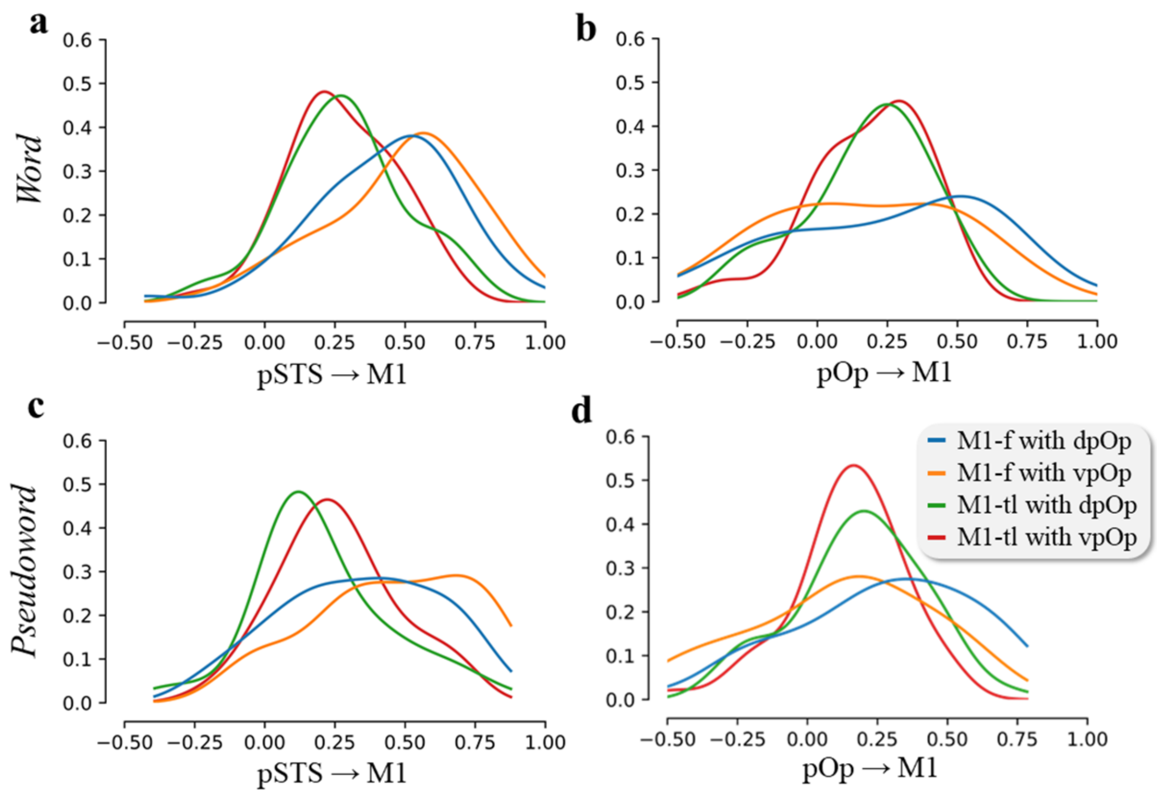

Supplementary Figure 1. Individual-level effective connectivity from pOp and pSTS to M1. The two panels in the first row (a) summarise connections across the different subregional configurations for word repetition, and the two panels in the second row (b) summarise connections across the different subregional configurations for pseudoword repetition: blue (M1-f and dpOp), orange (M1-f and vpOp), green (M1-tl and dpOp) and red (M1-tl and dpOp). The first panel in each row presents the sample density for individual connections from pSTS to M1 and the second panel in each row presents the sample density over the connections from pOp to M1.

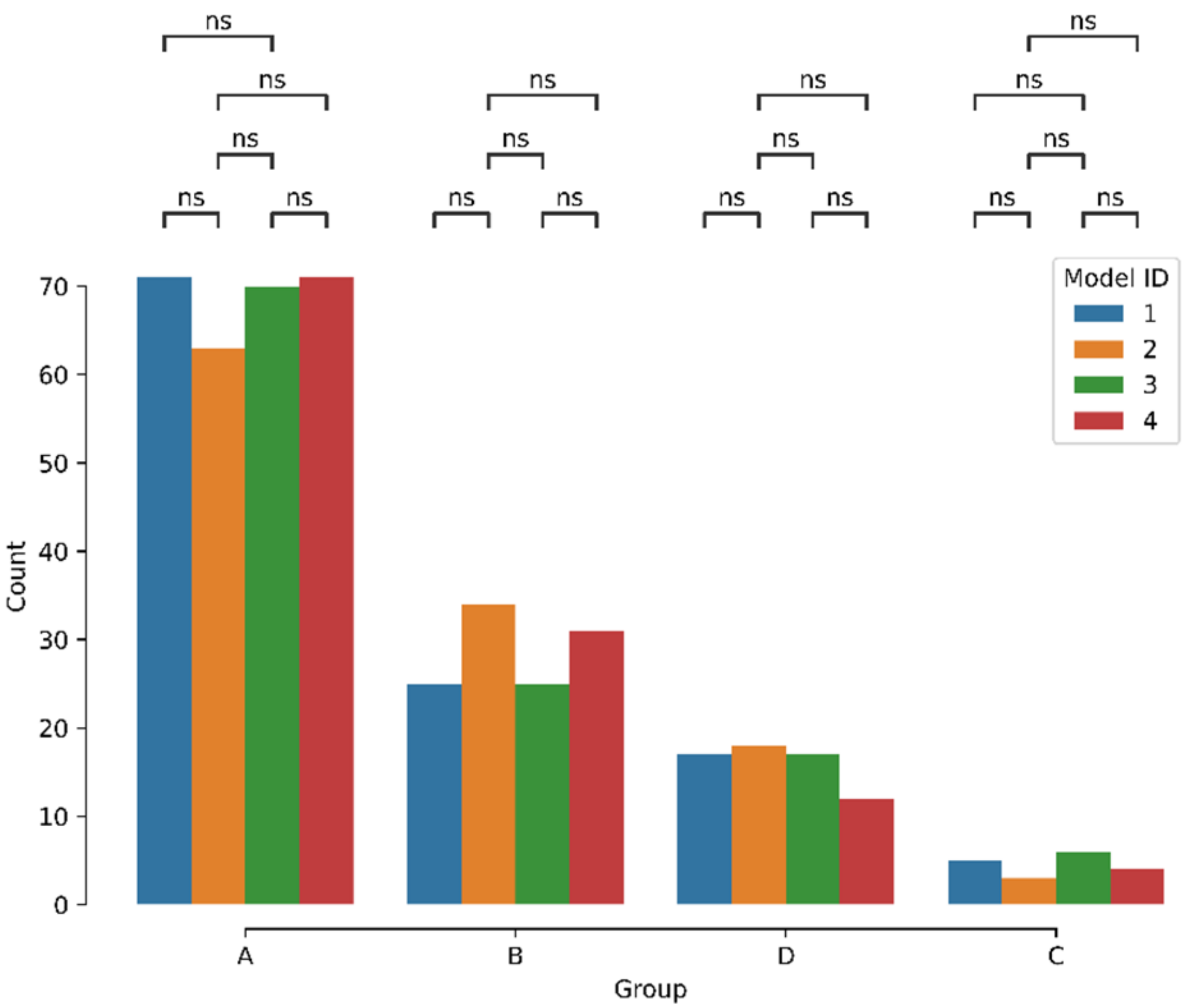

Supplementary Figure 2. Variability in group membership. The bar plot reports the differences in group membership across the different model configurations: 1 (M1-f and dpOp), 2 (M1-f and vpOp), 3 (M1-tl and dpOp) and 4 (M1-tl and dpOp). Here, the x-axis is the group, and the y-axis reports the total number of models in that group. We found no significant differences (with p-value of 1.00) between word and pseudoword repetition using a two-sided Mann-Whitney-Wilcoxon test with Bonferroni correction.

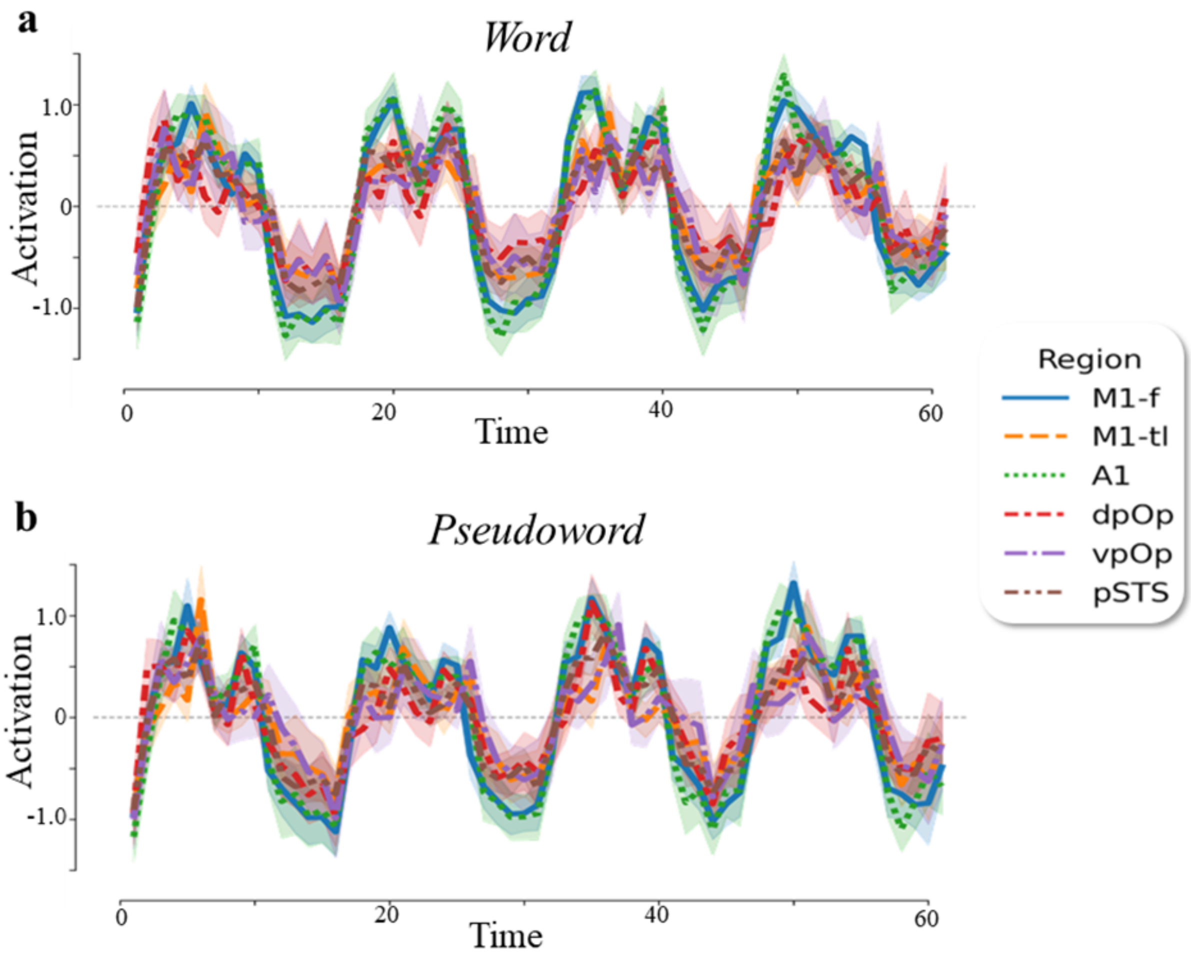

Supplementary Figure 3. Activation in each subregion of interest (ROI) during word and pseudoword repetition. The plots indicate activation in each ROI over the trial duration during word (a) and pseudoword repetition (b). Specifically, x-axis is the first principal component of the pre-whitened, high-pass filtered and confounded corrected timeseries for each ROI. The y-axis plots brain activation in each region: M1-f (blue), M1-tl (orange), dpOp (red) vpOp (purple), pSTS (brown) and A1 (green). Here, the shading represents the represents the 95% confidence interval.

Supplementary Table 1. Summary of participant data by group, for each word repetition DCM subregional configuration. Here, 1-4 denotes the different subregional configurations: 1 (M1-f and

dpOp), 2 (M1-f and vpOp), 3 (M1-tl and dpOp) and 4 (M1-tl and dpOp). Here, A had positive connections from pOp to M1 and pSTS to M1; B had positive connections from pSTS to M1 but not from pOp to M1; C had positive connections from pOp to M1 but not from pSTS to M1, and D denotes the posterior probability of < 0.75 i.e., no significant connections from both pOp and pSTS to M1. Additionally, C* denotes consistency with the neurological model.

				**Word Repetition**		**Pseudoword Repetition**	
	**Group**	**No. Subjects**	**Age**	**Accuracy (%)**	**Reaction Time (msec)**	**Accuracy (%)**	**Reaction Time (msec)**
**1**	A	35	44.42	99.64 (+/- 1.07)	1191.84 (+/- 164.79)	97.00 (+/- 4.77)	1278.05 (+/- 239.03)
	B	15	41.37	100.00 (+/- 0.00)	1148.71 (132.30)	99.67 (+/- 0.88)	1210.37 (+/- 108.77)
	C*	2	44.64	100.00 (+/- 0.00)	1125.95 (+/- 148.54)	100.00 (+/- 0.00)	1380.79 (+/- 210.77)
	D	7	42.43	97.86 (+/- 2.25)	1085.01 (+/- 193.90)	97.14 (+/- 6.52)	1154.28 (+/- 147.66)
**2**	A	32	47.03	99.38 (+/- 1.42)	1155.74 (+/- 193.66)	97.19 (+/- 4.91)	1247.64 (+/- 235.89)
	B	18	41.23	99.72 (+/- 1.18)	1168.46 (+/- 123.09)	98.61 (+/- 4.13)	1238.10 (+/- 184.86)
	C	1	31.10	100.00	1211.35	100.00	1234.91
	C*	1	29.63	97.50	1106.94	100.00	1230.91
	D	7	36.51	100.00 (+/- 0.00)	1208.24 (92.52)	97.86 (+/- 3.04)	1293.28 (+/- 135.76)
**3**	A	38	42.04	99.61 (+/- 1.09)	1150.70 (+/- 169.06)	98.22 (+/- 3.76)	1244.20 (+/- 230.28)
	B	9	47.49	99.72 (+/-0.83)	1204.40 (+/- 166.39)	96.39 (+/- 6.14)	1285.56 (+/- 162.79)
	C	2	34.81	97.50 (+/- 3.54)	1088.61 (+/- 12.61)	98.75 (+/- 1.77)	1170.32 (+/- 15.39)
	C*	1	23.38	100.00	1220.38	95.00	1219.60
	D	9	49.16	99.44 (+/- 1.67)	1203.15 (+/- 143.32)	97.50 (+/- 5.73)	1257.68 (+/- 170.26)
**4**	A	36	43.45	99.72 (+/- 1.00)	1147.58 (+/- 145.15)	98.47 (+/- 3.55)	1232.81 (+/- 199.43)
	B	4	47.64	100.00 (+/- 0.00)	1252.68 (+/- 104.01)	100.00 (+/- 0.00)	1320.26 (+/- 111.12)
	C*	1	43.50	100.00	1374.60	100.00	1349.46
	D	18	42.16	99.03 (+/- 1.74)	1171.88 (+/- 193.53)	95.83 (+/- 5.82)	1262.08 (+/- 237.75)

Supplementary Table 2. Summary of participant data by group, for each pseudoword repetition DCM subregional configuration. Here, 1-4 denotes the different subregional configurations: 1 (M1-f and

dpOp), 2 (M1-f and vpOp), 3 (M1-tl and dpOp) and 4 (M1-tl and dpOp). Here, A had positive connections from pOp to M1 and pSTS to M1; B had positive connections from pSTS to M1 but not from pOp to M1; C had positive connections from pOp to M1 but not from pSTS to M1, and D denotes the posterior probability of < 0.75 i.e., no significant connections from both pOp and pSTS to M1. Additionally, C* denotes consistency with the neurological model.

				**Word Repetition**		**Pseudoword Repetition**	
	**Group**	**No. Subjects**	**Age**	**Accuracy (%)**	**Reaction Time (msec)**	**Accuracy (%)**	**Reaction Time (msec)**
1	A	36	42.94	99.65 (+/- 1.06)	1178.74 (+/- 149.45)	97.71 (+/- 4.45)	1268.73 (+/- 177.67)
	B	10	40.17	99.00 (+/- 1.75)	1164.90 (+/- 137.10)	97.25 (+/- 3.99)	1250.54 (+/- 158.21)
	C*	3	29.24	100.00 (+/- 0.00)	1144.28 (+/- 126.49)	100.00 (+/- 0.00)	1348.79 (+/- 308.33)
	D	10	54.72	99.50 (+/- 1.58)	1127.54 (+/- 232.88)	98.00 (+/- 5.50)	1150.30 (+/- 292.76)
2	A	31	42.34	99.44 (+/- 1.40)	1184.52 (+/- 139.66)	97.58 (+/- 4.98)	1281.07 (+/- 183.83)
	B	16	44.17	99.53 (+/- 1.36)	1154.59 (+/- 129.05)	97.19 (+/- 4.46)	1211.30 (+/- 197.73)
	C	1	29.10	100.00	1418.15	92.50	1363.33
	D	11	46.75	99.77 (+/- 0.75)	1107.30 (+/- 233.21)	99.77 (+/- 0.75)	1206.53 (+/- 272.98)
3	A	32	42.41	99.38 (+/- 1.42)	1145.11 (+/- 146.47)	98.05 (+/- 4.29)	1235.92 (+/- 211.78)
	B	8	38.38	99.69 (+/- 0.88)	1189.39 (+/- 127.74)	97.81 (+/- 4.11)	1281.69 (+/- 169.49)
	C	1	55.07	100.00	1300.55	97.50	1341.12
	C*	2	33.91	100.00 (+/- 0.00)	1309.76 (+/- 139.18)	100.00 (+/- 0.00)	1380.48 (+/- 205.86)
	D	16	48.63	99.69 (+/- 1.25)	1169.58 (+/- 202.72)	97.03 (+/- 5.26)	1239.00 (+/- 222.18)
4	A	35	40.36	99.43 (+/- 1.37)	1137.46 (+/- 178.80)	98.14 (+/- 4.26)	1202.15 (+/- 227.17)
	B	8	43.68	99.69 (+/- 0.88)	1245.40 (+/- 98.32)	96.25 (+/- 5.35)	1392.60 (+/- 167.18)
	C	3	47.78	100.00 (+/- 0.00)	1279.54 (+/- 111.47)	100.00 (+/- 0.00)	1403.16 (+/- 150.77)
	D	13	52.20	99.62 (+/- 1.39)	1167.62 (+/- 127.77)	97.31 (+/- 4.73)	1254.11 (+/- 101.57)
