# Supplementary material for: Degeneracy in the neurological model of auditory speech repetition
Source: Commun Biol. 2023 Nov 13;6:1161. doi: 10.1038/s42003-023-05515-5 (PMC10643365; doi:10.1038/s42003-023-05515-5)
Supplement: Supplementary file 3 — Description of Additional Supplementary Files [file 42003_2023_5515_MOESM3_ESM.pdf]

Description of Additional Supplementary Files

File name: Supplementary Data 1
Description: The source data for participants group assigned across different model configuration.

File name: Supplementary Data 2
Description: The source data behind the graphs in Figure 3 and Figure 4

File name: Supplementary Data 3
Description: The source data behind the graphs in Figure 3
