# Supplementary material for: Degeneracy in the neurological model of auditory speech repetition
Source: Commun Biol. 2023 Nov 13;6:1161. doi: 10.1038/s42003-023-05515-5 (PMC10643365; doi:10.1038/s42003-023-05515-5)
Supplement: Supplementary file 7 — Reporting Summary [file 42003_2023_5515_MOESM7_ESM.pdf]

nature portfolio

Corresponding author(s): Noor Sajid

Last updated by author(s): Oct 4, 2023

Reporting Summary

Nature Portfolio wishes to improve the reproducibility of the work that we publish. This form provides structure for consistency and transparency in reporting. For further information on Nature Portfolio policies, see our Editorial Policies and the Editorial Policy Checklist.

Statistics

For all statistical analyses, confirm that the following items are present in the figure legend, table legend, main text, or Methods section.

n/a	Confirmed	
☐	☒	The exact sample size (n) for each experimental group/condition, given as a discrete number and unit of measurement
☐	☒	A statement on whether measurements were taken from distinct samples or whether the same sample was measured repeatedly
☐	☒	The statistical test(s) used AND whether they are one- or two-sided<br>*Only common tests should be described solely by name; describe more complex techniques in the Methods section.*
☐	☒	A description of all covariates tested
☒	☐	A description of any assumptions or corrections, such as tests of normality and adjustment for multiple comparisons
☒	☐	A full description of the statistical parameters including central tendency (e.g. means) or other basic estimates (e.g. regression coefficient) AND variation (e.g. standard deviation) or associated estimates of uncertainty (e.g. confidence intervals)
☐	☒	For null hypothesis testing, the test statistic (e.g. F, t, r) with confidence intervals, effect sizes, degrees of freedom and P value noted<br>*Give P values as exact values whenever suitable.*
☐	☒	For Bayesian analysis, information on the choice of priors and Markov chain Monte Carlo settings
☒	☐	For hierarchical and complex designs, identification of the appropriate level for tests and full reporting of outcomes
☐	☒	Estimates of effect sizes (e.g. Cohen's d, Pearson's r), indicating how they were calculated

Our web collection on statistics for biologists contains articles on many of the points above.

Software and code

Policy information about availability of computer code

Data collection	Functional MRI (fMRI) data were acquired on a 3T Trio scanner (Siemens Medical Systems) using a 12-channel head coil and a gradient-echo EPI sequence with 3 × 3 mm in-plane resolution (repetition time/echo time/flip angle: 3080 ms/30 ms/90°, extended field of view = 192 mm, matrix size = 64 × 64, 44 slices, slice thickness = 2 mm, and interslice gap = 1 mm). Structural MRI data were high-resolution T1-weighted images, acquired on the same 3T scanner using a 3D modified driven equilibrium Fourier transform sequence 45: TR/TE/TI = 7.92 ms/2.48 ms/910 ms, Flip angle = 16, 176 slices, voxel size = 1x1x1 mm3.
Data analysis	All data processing and analyses were performed with the Statistical Parametric Mapping (SPM12) software package (Wellcome Centre for Human Neuroimaging, London UK; http://www.fil.ion.ucl.ac.uk/spm/). All functional volumes were spatially realigned, unwarped, normalised to MNI space using a standard normalisation-segmentation procedure, and smoothed with a 6 mm full-width half-maximum isotropic Gaussian kernel, with a resulting voxel size of 3 × 3 × 3 mm. The unwarping step corrects for distortions caused by head movement or magnetic field inhomogeneity. Within each scanning run, all participant's movements, were less than one voxel (3 × 3 × 3 mm).<br>The first level (fixed effects) analysis used the general linear model (GLM) to fit the pre-processed functional volumes for each of the 13 conditions (including word and pseudoword repetition). Separate regressors were entered for instructions, correct responses, incorrect responses and "other" responses (delayed, no response, or self-corrected). Each stimulus onset was modelled as a single event within each regressor. The contrasts of interest were those that modelled correct responses for word repetition compared to resting fixation and correct responses for pseudoword word repetition compared to resting fixation.

For manuscripts utilizing custom algorithms or software that are central to the research but not yet described in published literature, software must be made available to editors and reviewers. We strongly encourage code deposition in a community repository (e.g. GitHub). See the Nature Portfolio guidelines for submitting code & software for further information.

Data

Policy information about availability of data

All manuscripts must include a data availability statement. This statement should provide the following information, where applicable:

- Accession codes, unique identifiers, or web links for publicly available datasets
- A description of any restrictions on data availability
- For clinical datasets or third party data, please ensure that the statement adheres to our policy

Data is available via request to c.j.price@ucl.ac.uk

Human research participants

Policy information about studies involving human research participants and Sex and Gender in Research.

Reporting on sex and gender	Biological sex information was reported; 34 females and 25 males.
Population characteristics	We collected information on age, gender, right-handedness, reported vision and hearing, neurological condition and language ability.
Recruitment	All participants gave written informed consent before participation and were compensated £10 per hour for their time.
Ethics oversight	The study was approved by the London Queen Square Research Ethics Committee.

Note that full information on the approval of the study protocol must also be provided in the manuscript.

Field-specific reporting

Please select the one below that is the best fit for your research. If you are not sure, read the appropriate sections before making your selection.

☐ Life sciences ☒ Behavioural & social sciences ☐ Ecological, evolutionary & environmental sciences

For a reference copy of the document with all sections, see nature.com/documents/nr-reporting-summary-flat.pdf

Behavioural & social sciences study design

All studies must disclose on these points even when the disclosure is negative.

Study description	Quantitative analysis
Research sample	59 healthy subjects participated in our study [25 males and 34 females; mean age ± SD = 44.5 ± 17.66 years].
Sampling strategy	Subjects were recruited via advertisements for participation in an fMRI language study; PLORAS (https://www.ucl.ac.uk/ploras/ploras-predicting-language-outcome-and-recovery-after-stroke).
Data collection	We collected fMRI and structural MRI for each participant.
Timing	First inclusion fMRI: March 2016<br>Last inclusion fMRI: December 2019
Data exclusions	All participants were native English speakers, right-handed (assessed with the Edinburgh handedness inventory) neurologically intact and reported normal or corrected-to-normal vision and hearing.
Non-participation	No drop-outs
Randomization	All participants performed all the experiments. Crucially, the order of all conditions, the content of the stimuli and the presentation parameters were identical for all participants.

Reporting for specific materials, systems and methods

We require information from authors about some types of materials, experimental systems and methods used in many studies. Here, indicate whether each material, system or method listed is relevant to your study. If you are not sure if a list item applies to your research, read the appropriate section before selecting a response.

Materials & experimental systems

n/a	Involved in the study
☒	☐ Antibodies
☒	☐ Eukaryotic cell lines
☒	☐ Palaeontology and archaeology
☒	☐ Animals and other organisms
☒	☐ Clinical data
☒	☐ Dual use research of concern

Methods

n/a	Involved in the study
☒	☐ ChIP-seq
☒	☐ Flow cytometry
☐	☒ MRI-based neuroimaging

Magnetic resonance imaging

Experimental design

Design type: Block design

Design specifications: In each scanning run of 3.4 minutes, 40 words or pseudowords were presented sequentially with 4 blocks of 10 stimuli (25 seconds per block) interspersed with 16 seconds of rest.

Behavioral performance measures: Reaction time and accuracy.

Acquisition

Imaging type(s): Structural, Functional

Field strength: 3T Trio scanner

Sequence & imaging parameters: Functional MRI (fMRI) data were acquired on a 3T Trio scanner (Siemens Medical Systems) using a 12-channel head coil and a gradient-echo EPI sequence with 3 × 3 mm in-plane resolution (repetition time/echo time/flip angle: 3080 ms/30 ms/90°, extended field of view = 192 mm, matrix size = 64 × 64, 44 slices, slice thickness = 2 mm, and interslice gap = 1 mm).

Structural MRI data were high-resolution T1-weighted images, acquired on the same 3T scanner using a 3D modified driven equilibrium Fourier transform sequence 45: TR/TE/TI = 7.92 ms/2.48 ms/910 ms, Flip angle = 16, 176 slices, voxel size = 1x1x1 mm3.

Area of acquisition: Whole brain scan was used

Diffusion MRI: ☐ Used ☒ Not used

Preprocessing

Preprocessing software: All data processing and analyses were performed with the Statistical Parametric Mapping (SPM12) software package (Wellcome Centre for Human Neuroimaging, London UK; http://www.fil.ion.ucl.ac.uk/spm/).

Normalization: All functional volumes were spatially realigned, unwarped, and normalised.

Normalization template: Normalisation to MNI space using a standard normalisation-segmentation procedure, and smoothed with a 6 mm full-width half-maximum isotropic Gaussian kernel, with a resulting voxel size of 3 × 3 × 3 mm.

Noise and artifact removal: The unwarping step corrects for distortions caused by head movement or magnetic field inhomogeneity. Within each scanning run, all participant's movements, were less than one voxel (3 × 3 × 3 mm).

Volume censoring: N/A

Statistical modeling & inference

Model type and settings: Effective connectivity among four ROIs was estimated using dynamic causal modelling (DCM) as implemented in SPM12.

Effect(s) tested: We used two model parameters: (i) input parameters that identify which region was responding to external stimuli, here the primary auditory cortex; and (ii) the effective connectivity changes that occur among regions, as participants alternate between repetition and rest. These parameters were estimated at the neuronal level and the coupling between regions does not necessarily reflect the existence of direct connections.

Specify type of analysis: ☐ Whole brain ☒ ROI-based ☐ Both

Anatomical location(s)

The region of interest (ROI) selection process involved two steps. First, we defined the anatomical boundaries of each of our four regions of interest using the Brainnetome atlas 11 (Figure 1a). We selected regions Te1.0 and Te1.2 for the primary auditory cortex (A1), the rostro-posterior STS subregion for Wernicke's area (pSTS), the dorsal pOp subregion for Broca's Area (dpOp), and the face (including the mouth) subregion for the primary motor cortex (M1-f). These choices were guided by the fMRI findings from an independent group of 25 neurologically intact participants who performed the same word and pseudoword repetition tasks as reported in Hope et al. (2014). Additionally, we evaluated different parts of M1 and pOp due to (i) spatially extensive activation in these regions during auditory word and pseudoword repetition and (ii) lack of knowledge as to which parts of pOp were driving M1 and conversely which part of M1 was driven by pOp (or pSTS). For pOp, we exchanged the dorsal pOp subregion with the ventral pOp subregion (vpOp). For M1, we exchanged the face motor control subregion with the tongue and larynx motor control subregion (M1-tl) from the Brainnetome atlas. This resulted in 4 different (sub)regional configurations per subject for both word and pseudoword repetition (8 configurations per subject in total). We did not investigate different parts of A1 or pSTS because (i) we had strong a priori knowledge about the origin of auditory inputs in the primary auditory cortex and (ii) only the rostro-posterior part of STS was robustly activated by both word and pseudoword repetition. The region borders were determined using a probability threshold of 50%: i.e., the anatomical localisation of the regions was consistent for at least 50% of the neurologically intact participants who contributed to the atlas construction. These probability thresholds are within the range used in previous studies.

Second, we searched for the peak response during word and pseudoword repetition within each anatomically defined ROI in each of the 59 participants. Separate time series of activation during the word and pseudoword repetition tasks were extracted from the peak coordinates for each participant. This ensured that effective connectivity between regions was estimated where activation was most robust for each participant, within a given ROI.

Statistic type for inference
(See Eklund et al. 2016)

Voxel-wise; Dynamical Causal Model

Correction

Effects reported have been thresholded at voxel wise $p < 0.05$ FWE-corrected; Bayes factor > 3

Models & analysis

n/a	Involved in the study
☐	☒ Functional and/or effective connectivity
☒	☐ Graph analysis
☒	☐ Multivariate modeling or predictive analysis

Functional and/or effective connectivity

Pearson correlation, Bayes Factor, and Maximum Likelihood
